# Distribution of Staphylococcal cassette chromosome mecA (SCCmec) types among coagulase-negative *Staphylococci* isolates from healthcare workers in the North-West of Iran

**DOI:** 10.22038/ijbms.2020.48481.11127

**Published:** 2020-11

**Authors:** Zahra Shokravi, Mehdi Haseli, Laleh Mehrad, Ali Ramazani

**Affiliations:** 1 Department of Microbiology, Faculty of Basic Science, Islamic Azad University, Science and Research Branch of Tehran, Markazi, Iran; 2 Biotechnology Department, School of Pharmacy, Zanjan University of Medical Sciences, Zanjan, Iran

**Keywords:** Aminoglycosides modifying – enzymes, Coagulase-negative – Staphylococci, Healthcare workers, Methicillin resistance, Polymerase chain reaction

## Abstract

**Objective(s):**

Methicillin-resistant coagulase-negative *Staphylococci* (MR-CoNS) are recognized as one of the major causes of healthcare-associated infections in hospitals. The present investigation aimed to study the prevalence of Staphylococcal cassette chromosome mec (SCCmec) types, along with aminoglycoside modifying enzymes (AMEs) genes in the nasal carriage of MR-CoNS in the north-west of Iran.

**Materials and Methods:**

To assess the potential of coagulase-negative *Staphylococci* as hidden reservoirs for antibiotic resistance, we analyzed the antimicrobial susceptibility of MR-CoNS using the disk diffusion method. In addition, PCR and multiplex PCR assays were performed to determine the prevalence of AME encoding genes and SCCmec types in methicillin-resistant coagulase-negative *Staphylococci* isolates.

**Results:**

A total of 51 MR-CoNS isolates were recovered from the anterior nares of healthcare workers. The observed resistance rates to tobramycin, gentamicin, cotrimoxazole, kanamycin, erythromycin, tetracycline, and ciprofloxacin were 74.5%, 68.5%, 57%, 53%, 51%, 49%, and 8%, respectively. Of the 51 tested MR-CoNS isolates, 2(4%) were harboring *SCCmec* type I, four (8%) were type II, six (12%) type III, eleven (21.6%) type IVa, two (4%) type IVb, two (4%) type IVc, six (12%) type IVd, and two (4%) type V. The rates of prevalence of the aminoglycoside modifying enzyme genes were as follows: *aac (6′)/aph (2′′)* (28 cases, 55 %), ant *(4′)-Ia* (20 cases, 39%), and the *aph (3´)-IIIa* gene (9 cases, 17.6 %).

**Conclusion:**

Subtypes IVa and IVd were the most prevalent SCC elements, and aac (6′)/aph (2′′) was the most common AME gene detected among the MR-CoNS isolates.

## Introduction

*Staphylococci* are one of the most prevalent human pathogens. They are classified into coagulase-positive *Staphylococci*, *Staphylococcus aureus, *and coagulase-negative *Staphylococci* (CoNS)*. *Transmission of *Staphylococcus *species in hospital settings can increase the risk of invasive diseases ranging from minor skin infections to life-threatening illnesses (1). *Staphylococcus* species constitute nearly 30% of hospital-acquired infections and incur annual treatment costs of two billion dollars (2). Moreover, in recent decades, the rapid emergence of methicillin resistance in *Staphylococcus* species has posed a significant challenge that hampers the effective treatment of nosocomial infections. According to global surveillance studies, the frequency of MR-CoNS can reach up to 80% in hospital environments(3). Methicillin resistance in *Staphylococcus* species is primarily mediated by *the mecA *gene which is located on the mobile genetic element called SCC*mec *(4-6). Over the past few decades, CoNS isolates have not only acquired resistance to beta-lactam antibiotics but have also gained resistance against other antimicrobial agents. A number of these antibiotic-resistant genes (e.g., the genes that encode AMEs) are carried on the small plasmids and transposons that can be integrated into SCC*mec*. Therefore, these genetic elements are a source of resistance against genes that can be easily disseminated among *Staphylococci* strains (7-9).

MR-CoNS are transmitted by person-to-person contact, generally between patients and healthcare workers (HCWs). Therefore, investigating methicillin resistance in *Staphylococcus* strains in HCWs is of paramount importance in controlling the healthcare-associated infections in hospitals(10). As anterior nares are one of the major ecological niches for MR-CoNS and methicillin-resistant *S. aureus* (MRSA), nasal carriages of MR-CoNS and MRSA among HCWs can be a reliable indicator for determining the antibiotic-resistant pattern of *Staphylococci* strains in hospital environments (11, 12). Accordingly, a number of studies have been done on the nasal carriage of MRSA among HCWs in different parts of Iran (13-15)**.** However, the nasal carriage of MR-CoNS among HCWs is under-investigated. Pourmand *et al.* (16) showed high rates of *aap* and* mecA *genes in the nasal isolates of CoNS among HCWs in Iran. Khashei *et al.* (17) also reported a high prevalence of nasal carriage of MR-CoNS among nurses (67.4%) and non-patient care workers (20%) in six hospitals in Iran. Additionally, another study (18) on the prevalence of the nasal carriage of MR-CoNS among HCWs found higher carriage rates of MR-CoNS among nursing students (50%) than in other categories of HCWs. A similar study (19), reported a high prevalence of MR-CoNS carriage among doctors (41.6%) and nurses (32.4%) in India.

However, most of this research discusses only the nasal colonization of MR-CoNS and does not consider the molecular epidemiology of MR-CoNS isolated from nasal specimens. In addition, there are very few investigations on the simultaneous distribution of genes encoding AMEs and SCC*mec* types among nasal specimens of HCWs, particularly in Iran. In this respect, the colonization of MR-CoNS on the anterior nares of HCWs was studied in the present research. We also studied the distribution of SCC*mec* types and the most clinically important AMEs genes among MR-CoNS isolates from HCWs of two hospitals in northwest of Iran.

## Materials and Methods

***Isolation and the identification***

This cross-sectional study was carried out in two university teaching hospitals in Zanjan, Iran (Valiasre and Ayatollah Mousavi Hospitals), from March 2017 to January 2018. A total of 134 CoNS samples were isolated from HCWs who were working in different units of hospitals. Only one isolate per subject was included in this research. In addition, information pertaining to participants’ medical histories, work experiences, demographic information, and any other factors that could be related to the nasal carriage of MR-CoNS were collected through self-administered questionnaires. This study was approved by the Ethical Committee of Zanjan University of Medical Sciences (ethical code: 12/90-349-8). CoNS isolates were collected from nasal cavities with collection swabs and were directly inoculated onto mannitol salt agar and blood agar for 24 hr at 37 °C. 

Bacterial colonies morphologically consistent with CoNS were identified by morphological and biochemical tests, including the production of coagulase, oxidase, catalase, gram staining, DNase, and fermentation of mannitol. 

***PCR and multiplex PCR amplification***

All confirmed species were subjected to DNA extraction by the phenol/chloroform method. The genomic DNA was quantified using spectrophotometry and used as templates for detecting the *mecA* gene, AME genes, and SCC*mec *typing(19).

The *mecA *gene was detected by polymerase chain reactions (PCRs) as previously described (20). Briefly, the PCR conditions were as follows: initial denaturation at 92 °C for 20 sec, 30 cycles of denaturation at 94 °C for 40 sec, annealing at 58 °C for 20 sec, extension at 72 °C for 20 sec, and a final extension at 72 °C for 7 min. The bacterial isolates that were detected for 310 bp amplicon were characterized as MR-CoNS. 

SCC*mec *typing was performed using multiplex PCR as previously described (21). In this study, *S. aureus* strains RM911, RM912, RM913, RM914, and RM917 were used as positive controls for *SCCmec *types I, II, III, IV, and V, respectively. 

Additionally, three sets of primers specific for *ant (4*′*)-Ia*, *aph (3*′*)-IIIa* and* aac (6*′*)/aph (2*′′*)* were selected from published sequences(22).  All MR-CoNS strains were analyzed for the presence of *ant (4*′*)-Ia*, *aph (3’)-IIIa* and *aac (6’)-Ie-aph (2”)-Ia* genes with PCR master mix (Fermentas, K01071) containing 10 pM for each primer, 500 ng genomic DNA, and distilled water to a final volume of 25 μl.* Enterococcus faecalis* HH22 and *Enterococcus faecalis* JH2-2 were used as positive and negative controls, respectively. The PCR reactions were carried out in the thermocycler (iCycler, BIO-RAD). After amplification, PCR products were subjected to electrophoresis in 1.5% agarose. The agarose gels were stained with Syber Green, DNA staining dye, and then visualized under ultraviolet light. 

***Antimicrobial susceptibility testing***

Antimicrobial susceptibility profiles of MR-CoNS were determined based on guidelines provided by the Clinical & Laboratory Standards Institute )CLSI(. The antibiotic disks used were penicillin G (10 mg), oxacillin (1 μg), tobramycin (10 mg), gentamicin (30 μg), kanamycin (30 μg), cotrimoxazole (1.25 μg), tetracycline (30 μg)**,** ciprofloxacin (5 μg), erythromycin (15 μg), and vancomycin (30 μg). *S. aureus* (ATCC 29213) was used as a susceptible control strain at each set of tests. The results of antimicrobial susceptibility profiles were interpreted according to the CLSI breakpoints(23, 24). An intermediate susceptibility level was considered as resistant (25).

***Data analysis***

The statistical analyses were performed with SPSS (version 16.0 for Windows; SPSS Inc., Chicago, IL). Fisher’s exact or Chi-square test was used for the analysis of data. A *P-value* of < 0.05 was considered statistically significant.

## Results

A total of 168 non-duplicate nasal specimens were recovered from HCWs. *Staphylococci* were found in 161 (96%) of 168 specimens. Of the 161 *Staphylococci* samples, 134 (83%) were identified as CoNS strains. In addition, the *mec*A gene was found in 51 (38%) of CoNS isolates. Among them, 8/53% were women and 46% were men. The average age of the studied population was 37±12 years. Nearly half of the participants had less than ten years of work experience. There was no significant difference between carriage and non-carriage of MR-CoNS in terms of work experience. 

**Figure 1:**
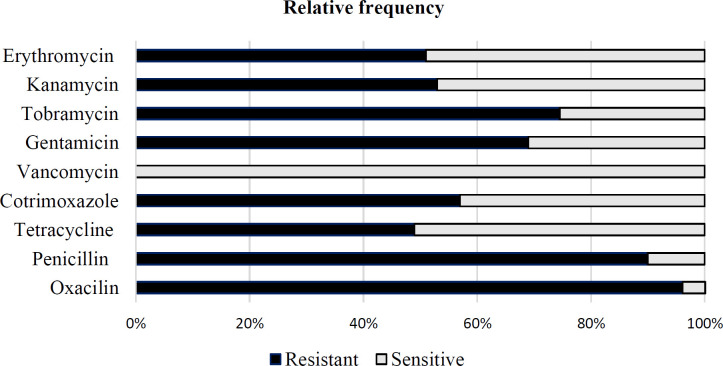
Antimicrobial resistance rates in MR-CoNS isolates

Participants who had taken antibiotics during the two months prior to the study had a lower rate of MR-CoNS carriage than those who had not. The antibiotic susceptibility patterns of 51 nasal carriage strains of MR-CoNS are shown in Figure 1.

The highest percentages of MR-CoNS resistance were to oxacillin 49 (96%), penicillin 46(90%), cotrimoxazole29 (57%), erythromycin 26 (51%), tetracycline 25 (49%), and ciprofloxacin 4(8%). Regarding the phenotypic resistance patterns of the MR-CoNS strains to aminoglycosides, 38 (74.5%) were resistant to tobramycin, 35 (68.5%) to gentamicin, and 27 (53%) to kanamycin. All MR-CoNS isolates were sensitive to vancomycin. Multi-drug-resistant isolates that were resistant to more than three antimicrobial agents were detected in 29 (57%) isolates.

Among the studied population, type IVa (11cases*,* 21.6%) was the most frequent SCC*mec *type. Accordingly, 35 isolates (68.5%) had a single SCC*mec *type, as follows: type I (n=2), type II (n=4), type III (n=6), type IVb (n=2), type IVc (n=2), type IVd (n=6) and type V (n = 2). Overall, five isolates (10%) were untypeable. Additionally, eleven isolates had two SCC elements including III + V (n=2), II + V (n=3), II + I (n=3), and III + IVa (n=3). 

The prevalence rate of the AME genes was as follows: *aac (6′)/aph (2′′) *(28 cases, 55 %),* ant (4*′*)-Ia* (20 cases, 39%), and the *aph (3´)-IIIa *was the least prevalent AME gene identified in our study

(9 cases, 17.6%). Forty-one (80%) isolates carried one of these genes either alone or in combination with other genes. Two resistance genes of *aph (3*´*)-III *and* aac (6*′*)/aph (2*′′*) *were simultaneously presented in eight 8 (16%) of the isolates. 

## Discussion

Data for the Staphylococcal carriage of HCWs is important in determining the evolutionary potential of various antibiotic resistance genes in hospitals. While most current epidemiological surveillances concentrate on MRSA detection, research on nasal carriages of MR-CoNS between HCWs has remained relatively scarce. 

In the current study, the frequency of methicillin resistance among CoNS was found to be 38%. This resistance rate was lower than the previous studies from Iran but approximately similar to research from other countries. Khashei *et al.* (17) studied the prevalence of the nasal carriage of MR-CoNS among HCWs and found a 67.4% carriage rate among nurses. Pourmand *et al.* (16), reported *mecA *gene prevalence of 96%, which is higher than the data presented in this research. Consistent with our results, MR-CoNS carriage rate of 41% among HCWs was reported from India (19). 

Furthermore, the phenotypic resistance of MR-CoNS to different antibiotic classes was studied. In this investigation, the maximum resistance of MR-CoNS nasal specimens was observed towards beta-lactam antibiotics, followed by cotrimoxazole (57%), erythromycin (51%), and tetracycline (49%). This antibiotic resistance rate was basically lower than other studies from Iran(26, 27), but was similar to the previous studies from other countries (18). 

57% of our MR-CoNS isolates were resistant to three or more of the studied non-beta lactam antibiotics. The high prevalence of multi-drug-resistant strains is a crucial issue in hospitals because it represents a loss of the efficiency of available antibiotics toward pathogenic *Staphylococci* strains (Table 1).

In this investigation, SCC*mec *typing was used for epidemiological analysis of MR-CoNS isolates. As described in previous reports, the prevalence of SCC*mec *types in MR-CoNS varies depending on the geographical location and host species. Different SCC*mec *types in MR-CoNS have been distributed in various countries. For instance, types II, III, and V have been reported as the most frequent SCC elements in southern China(28), whereas SCC*mec *type I (50%) was the most common SCC*mec *type among MR-CoNS in India (29). However, limited information is available about various SCC*mec *types among MR-CoNS in Iran. A study (30) showed that SCC*mec *IV was the most frequent SCC*mec *type among MR-CoNS recovered from clinical specimens. Another report indicated that SCC*mec* IV and V were dominant in nasal specimens in children (31). In the current study, subtypes of IVa and IVd were dominant among MR-CoNS strains, which is consistent with previous works (30-32). However, in contrast to Halaji *et al.* (27), who reported that type I was the most prevalent SCC element among MR-CoNS isolates in central Iran, type I was very rare in our study.

The emergence of a large number of MR-CoNS strains harboring SCC*mec *IV is currently of great concern in terms of antibiotic resistance (33).

In this study, high rates of resistance to non-β-lactam antibiotics were observed among SCC*mec *type IV positive isolates. Moreover, around 42% of isolates that belonged to SCC*mec* type IV carried AME genes. Meanwhile, SCC*mec *type III was relatively frequent in our MR-CoNS isolates. In this investigation, five of six isolates that belonged to SCC*mec* III possessed at least one of the studied AME genes (Table 2). This higher rate might be due to the larger size of type III, which makes it suitable for carrying antibiotic-resistant genes (34).

Several strains observed in this study (n=11) had more than one SCC*mec *element. This is not surprising because the existence of more than one SCC*mec *type is common among MR-CoNS strains. 

In addition, few of the MR-CoNS isolates (n = 5) that were recovered in this research consisted of untypeable SCC elements. The presence of these untypeable isolates, as well as strains with more than a SCC*mec *element, indicates the development of new variants of SCC*mec* in the hospital environment. This is alarming for the development of new antibiotic-resistant *Staphylococci* in hospitals (35).

Aminoglycoside antibiotics play a major role in treating serious Staphylococcal infections despite the reports of increasing resistance to these antibiotics (22, 35, 36). Aminoglycoside inactivation by AME genes is considered the major resistance mechanism(22). Aminoglycoside resistance genes were found in 41 (80%) of the MR-CoNS isolates studied in this work. The majority of the isolates that possessed AME genes were resistant to aminoglycosides. Additionally, we found that 9 (17.6%) of the strains that were resistant to at least one of the aminoglycosides had no AME genes. This result corroborates that reported by other researchers (22). This could be due to other resistance mechanisms, such as amino acid alteration in ribosomal proteins or the decreased permeability of the antibiotics.

In consistent with many other findings (35, 37, 38), the *aac (6′)/aph (2′′) gene* was the most common AME gene found in this study; it was present in 55% of the isolates observed. The frequency rate of the *aac (6′)/aph (2′′) gene* in the present study was considerably lower than those of the other studies in Iran(37) and higher than a study from Turkey (39). These different results originated from regional differences in antibiotic use policies. 

In this research, the correlation between the presence of the *aac (6′)/aph (2′′) gene* and resistance to gentamicin and kanamycin was 86% and 71%, respectively. However, the correlation between the presence of *aac (6′)/aph (2′′)* gene and tobramycin resistance was less than 53%.

The second-most frequent AME gene detected in this study was the *ant (4′)-Ia gene* (39%). This is fairly similar to reports from other countries (22) but higher than a previous study from Iran (28%) (37).

The *aph (3´)-IIIa *gene was the least common gene detected in this study, which mediates resistance to kanamycin. In the current research, the concordance between the presence of the *aph (3´)-IIIa *gene and kanamycin resistance was 66%. 

## Conclusion

In this investigation, a high rate of resistance to multiple classes of antibiotics was observed among the MR-CoNS isolates. The function of CoNS as putative reservoirs for antibiotic resistance is still an underestimated issue that needs to be investigated. In this respect, having information about the aminoglycoside resistance epidemiology can save the aminoglycosides for the treatment strategies and limit the emergence of resistant Staphylococcal species in the hospital environment. Meanwhile, regarding the role of the SCC*mec* elements as the reservoir of virulence factors, the present study provides information that can be beneficial in future investigations.
